# Diagnostic and Predictive Value of Immune-Related Genes in Crohn’s Disease

**DOI:** 10.3389/fimmu.2021.643036

**Published:** 2021-04-16

**Authors:** Bing Yu, Yi-xin Yin, Yan-ping Tang, Kang-lai Wei, Zhi-gang Pan, Ke-Zhi Li, Xian-wen Guo, Bang-li Hu

**Affiliations:** ^1^ Department of Gastroenterology, Second Affiliated Hospital of Guangxi Medical University, Nanning, China; ^2^ Department of Research, Guangxi Medical University Cancer Hospital, Nanning, China; ^3^ Department of Gastroenterology, Third Affiliated Hospital of Guangxi Medical University, Nanning, China; ^4^ Department of Gastroenterology, People’s Hospital of Guangxi Zhuang Autonomous Region, Nanning, China

**Keywords:** Crohn’s disease, immune-related genes, gene set enrichment analysis, protein-protein interaction, differentially expressed genes

## Abstract

Abnormal immune cell infiltration is associated with the pathogenesis of Crohn’s disease (CD). This study aimed to determine the diagnostic and predictive value of immune-related genes in CD. Seven Gene Expression Omnibus datasets that analyzed the gene expression in CD tissues were downloaded. Single-sample gene set enrichment analysis (ssGSEA) was used to estimate the infiltration of the immune cells in CD tissues. Immune-related genes were screened by overlapping the immune-related genes with differentially expressed genes (DEGs). The protein-protein interaction (PPI) network was used to identify key immune-related DEGs. Diagnostic value of CD and predictive value of anti-TNFα therapy were analyzed. Immunohistochemical (IHC) assay was used to verify gene expression in CD tissues. There were significant differences among CD tissues, paired CD tissues, and normal intestinal tissues regarding the infiltration of immune cells. *AQP9*, *CD27*, and *HVCN1* were identified as the key genes of the three sub-clusters in the PPI network. *AQP9*, *CD27*, and *HVCN1* had mild to moderate diagnostic value in CD, and the diagnostic value of *AQP9* was better than that of *CD27* and *HVCN1*. AQP9 expression was decreased in CD after patients underwent anti-TNFα therapy, but no obvious changes were observed in non-responders. *AQP9* had a moderate predictive value in patients who had undergone treatment. IHC assay confirmed that the expression of *AQP9*, *CD27*, and *HVCN1* in CD tissues was higher than that in normal intestinal tissues, and *AQP9*, *CD27* was correlated with the activity of CD. Immune-related genes, *AQP9*, *CD27*, and *HVCN1* may act as auxiliary diagnostic indicators for CD, and *AQP9* could serve as a promising predictive indicator in patients who underwent anti-TNF therapy.

## Introduction

Crohn’s disease (CD) is a major type of inflammatory bowel disease (IBD), affecting approximately 0.5% of the worldwide population, and the incidence rate is increasing in China (1, 2). CD is an immune-mediated disorder characterized by chronic inflammation in the gastrointestinal tract. The exact etiology of CD remains largely unknown, but genetic and environmental factors are believed to be the main contributors that trigger inflammatory processes in the intestinal mucosa (3, 4). To date, no curative medical approach is available for CD (5); therefore, exploring the role of immune cells and the related cytokines is crucial for the development of effective therapeutic strategies for patients with CD.

Previous studies have shown that abnormal changes in immune cells and their effective cytokines exaggerate immune responses in the intestinal mucosa of patients with CD. For instance, CD14^+^ macrophages participate in CD by inducing activation-induced cell death resistance in CD4^+^ T cells (6). CK2α contributes to the pathogenesis of CD by promoting CD4^+^ T cell proliferation and Th1 and Th17 response (7). Human leukocyte antigen class II molecules, IL-1, IL-6, and IL-8, are also overexpressed in the intestinal epithelial cells of patients with active CD (8). This evidence demonstrates that chronic inflammation is induced by immune cells in patients with CD.

With the advancement in genomic sequencing technology, an increasing number of microarray analyses of disease-related datasets have been conducted, and these datasets have become an ideal source to quantify immune cell infiltration in various diseases. Recently, Chen et al. (9) provided an overview of immune cell alterations in CD using Gene Expression Omnibus (GEO) datasets and found that CXCL8 and IL-1B have a good diagnostic value in CD. However, relevant cytokines still need to be elucidated. Therefore, in this study, we used single-sample gene set enrichment analysis (ssGSEA) aiming to estimate immune cell infiltration in CD, and assess the role of immune-related cytokines in CD, which will highlight the relationship between immune infiltration at the molecular level and provide potential therapeutic targets for patients with CD.

## Materials and Methods

### Microarrays Dataset Collection and Data Process

The microarray datasets that investigated the gene expression of CD tissues were downloaded from the GEO database, which included GSE95095 (24 CD tissues, 24 non-inflammatory tissues, and 12 normal intestinal tissues), GSE36807 (13 CD tissues and 13 normal intestinal tissues), GSE98820 (80 CD tissues that before and after adalimumab treatment), GSE112366 (362 CD tissues and 26 normal intestinal tissues), GSE16879 (73 CD tissues and 12 normal intestinal tissues), GSE10616 (32 CD tissues and 13 normal intestinal tissues), and GSE102133 (65 CD tissues and 12 normal intestinal tissues) datasets. These seven datasets comprised CD tissues, paired CD tissues, and normal intestinal tissues. The raw data were preprocessed *via* background adjustment, quantile normalization, final summarization, and log2 transformation, as previously described (9).

### Immune Infiltration Analysis for the Microarrays Datasets

The ssGSEA score was used to determine the level of immune infiltration in each sample of datasets, which was calculated according to the expression levels of immune cell-specific marker genes (10). By defining immune cell-related gene sets, the enrichment score of the gene set represented the density of tumor-infiltrating immune cells. The marker genes for 28 types of immune cells were obtained from a previously published article (11), which included 28 common immune cells and 782 genes. The ssGSEA analysis was performed using the R language (version 3.6.1).

### Identification of Immune-Related Differentially Expressed Genes (DEGs)

The probe in each dataset was firstly converted into gene symbol, when there were multiple probes mapped to the same gene symbol; we selected their mean value as the gene expression value. The DEGs between CD and paired CD tissues; or between CD and normal intestinal tissues were analyzed using the “limma package” in R language. Multiple testing corrections were performed using the Beniamini-Hochberg (HB) method. DEGs with a p-value less than 0.01 were considered significant. DEGs and DEGs overlapping with immune cell-specific marker genes were defined as immune-related DEGs, which as selected in the R language and visualized by Venn graph.

### Gene Ontology and Protein-Protein Interaction (PPI) Network Analysis of Immune-Related DEGs

Functions of immune-related DEGs were analyzed using Gene Ontology (GO) analyses. GO analyses included biological process (BP), molecular function (MF), and cellular component (CC). Kyoto Encyclopedia of Genes and Genomes (KEGG) analysis was used to identify the significant pathways for gene enrichment. The “clusterProfiler” package (12) was used to conduct the GO analysis, and the p value < 0.05 was considered statistically significant enrichment. The PPI network for the immune-related DEGs was constructed based on the data from the STRING database. STRING database is an online tool for the analysis of protein and protein interactions, which can be used to obtain unique, wide-ranging experimental confirmed, predictive interaction information, and the combined score indicated the interaction between two proteins. In this study, only the interaction pairs with a PPI combined score > 0.7 were selected as significant. A plug-in of Cytoscape, Molecular Complex Detection (MCODE) was also applied to identify the sub-clusters of the PPI network. The core gene of each sub-cluster was selected based on the scores of the genes in the sub-clusters (MCODE score >3; the number of nodes >5).

### Immunohistochemical (IHC) Assay

Formalin-fixed, paraffin-embedded tumor tissues were collected from 20 patients with CD diagnosed at the People’s Hospital of Guangxi Zhuang Autonomous Region from December 2019 to June 2020. This study was approved by the Ethics Committee of the People’s Hospital of Guangxi Zhuang Autonomous Region. Primary antibodies used were as follows: Polyclonal rabbit anti−AQP9 (cat. no. 862723; 1:100 dilution; Zen Bioscience); polyclonal rabbit anti−CD27 (cat. no. 383803; 1:100 dilution; Zen Bioscience); polyclonal rabbit anti−HVCN1 (cat. no. 14162-1-AP; 1:200 dilution; Proteintech). The IHC assay was conducted as previously reported (13). IHC results for AQP9, CD27, and HVCN1 were evaluated utilizing the intensity score, which was combined with the staining intensity and area. The intensity score ranged from 0 to 9, with 0 defining no expression and 9 defining high expression.

### Statistical Analysis

Mann–Whitney U-test or Student’s t-test was used to compare continuous variables in clinical features between the two groups when appropriate. The ANOVA method was applied to compare the continuous variables among the three groups. Correlation between gene expression and the fraction of immune cells was analyzed using Pearson analysis. The diagnostic value of gene expression in patients with CD and the predictive value of the gene in patients with CD who underwent treatment was analyzed using receiver operating characteristic curves, with the area under the curve (AUC) used to estimate the diagnostic or predicted value. All statistical analyses were performed using R software (version 3.6.0) and SPSS version 19.0 software (SPSS, Inc., Chicago, IL, USA). A two-tailed p-value < 0.05 was considered statistically significant.

## Results

### The Landscape of Immune Infiltration in Crohn’s Disease

**Figure 1:**
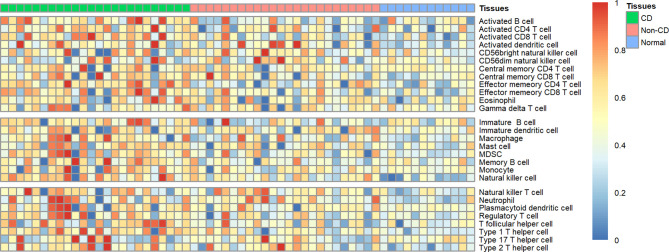
Heatmap of the landscape of 28 immune cell subpopulations infiltration in CD (n=60 tissues).

Using ssGSEA, we revealed the landscape of infiltration of 28 immune cell subpopulations in CD by analyzing the GSE95095 dataset. The fraction of immune cells varied distinctly among the three groups (**Figure 1**). Using the ANOVA method, we found that the fraction of 15/28 immune cells varied distinctly among these three kinds of tissues, and when comparing the CD tissues with non-inflammatory tissues, 22 out of 28 immune cells showed remarkable differences (**Suplementary Figure S1**). The fraction of immune cells was the highest in CD tissues compared with that in non-inflammatory tissues and normal intestinal tissues. Details of the immune cell fraction are presented in **Table 1**.

**Table 1:** Landscape of immune infiltration in Crohn’s disease.

Immune cells types	CD tissues	Adjacent CD tissues	Control	P value
Activated B cell	0.282 (0.073, 0.560)	0.148 (-0.007, 0.473)	0.214 (-0.031, 0.418)	0.003
Activated CD4 T cell	0.207 (0.046, 0.382)	0.169 (-0.043, 0.339)	0.137 (-0.045, 0.282)	0.015
Activated CD8 T cell	0.319 (0.130, 0.486)	0.234 (0.041, 0.432)	0.170 (0.049, 0.356)	<0.001
Activated dendritic cell	0.217 (0.143, 0.345)	0.221 (0.110, 0.349)	0.174 (0.131, 0.226)	0.009
CD56bright natural killer cell	0.261 (0.130, 0.327)	0.265 (0.219, 0.334)	0.230 (0.220, 0.294)	0.234
CD56dim natural killer cell	0.206 (0.101, 0.409)	0.242 (0.119, 0.435)	0.219 (0.163, 0.298)	0.576
Central memory CD4 T cell	0.487 (0.221, 0.582)	0.484 (0.348, 0.538)	0.476 (0.452, 0.506)	0.937
Central memory CD8 T cell	0.327 (0.152, 0.446)	0.308 (0.132, 0.454)	0.285 (0.202, 0.330)	0.146
Effector memory CD4 T cell	0.224 (0.089, 0.336)	0.160 (0.031, 0.271)	0.147 (0.074, 0.247)	<0.001
Effector memory CD8 T cell	0.427 (0.227, 0.561)	0.385 (0.250, 0.508)	0.361 (0.270, 0.422)	0.011
Eosinophil	0.073 (-0.120, 0.195)	0.056 (-0.124, 0.144)	0.052 (-0.168, 0.101)	0.337
Gamma delta T cell	0.178 (0.028, 0.272)	0.181 (-0.056, 0.242)	0.166 (0.117, 0.229)	0.915
Immature B cell	0.224 (-0.183, 0.403)	0.060 (-0.157, 0.492)	0.180 (-0.043, 0.290)	0.044
Immature dendritic cell	0.286 (0.025, 0.356)	0.285 (0.064, 0.366)	0.249 (0.192, 0.323)	0.913
Macrophage	0.128 (-0.064, 0.303)	0.080 (-0.001, 0.226)	0.068 (0.007, 0.152)	0.015
Mast cell	0.408 (0.201, 0.500)	0.370 (0.022, 0.463)	0.317 (0.241, 0.435)	0.072
MDSC	0.446 (0.160, 0.618)	0.376 (0.102, 0.553)	0.330 (0.288, 0.427)	0.011
Memory B cell	0.215 (-0.087, 0.511)	0.148 (-0.011, 0.305)	0.161 (0.075, 0.269)	0.045
Monocyte	0.477 (0.259, 0.598)	0.450 (0.209, 0.536)	0.440 (0.411, 0.480)	0.684
Natural killer cell	0.232 (0.041, 0.320)	0.196 (0.104, 0.263)	0.091 (0.044, 0.188)	<0.001
Natural killer T cell	0.191 (-0.005, 0.342)	0.182 (0.067, 0.296)	0.128 (0.072, 0.211)	0.127
Neutrophil	0.028 (-0.352, 0.355)	0.014 (-0.147, 0.304)	-0.116 (-0.210, 0.155)	0.018
Plasmacytoid dendritic cell	0.527 (0.257, 0.648)	0.494 (0.303, 0.608)	0.481 (0.462, 0.528)	0.811
Regulatory T cell	0.281 (-0.035, 0.574)	0.214 (0.016, 0.415)	0.205 (0.097, 0.267)	0.012
T follicular helper cell	0.284 (0.159, 0.345)	0.231 (0.160, 0.333)	0.222 (0.126, 0.279)	0.016
Type 1 T helper cell	0.170 (0.044, 0.236)	0.143 (-0.064, 0.202)	0.110 (0.063, 0.136)	0.002
Type 17 T helper cell	-0.087 (-0.194, 0.172)	-0.0713 (-0.182, 0.140)	-0.052 (-0.115, 0.014)	0.910
Type 2 T helper cell	0.102 (0.019, 0.209)	0.089 (-0.006, 0.252)	0.045 (-0.013, 0.117)	0.009

### Identification of Key Immune-Related Genes in CD Using the PPI Network

**Figure 2:**
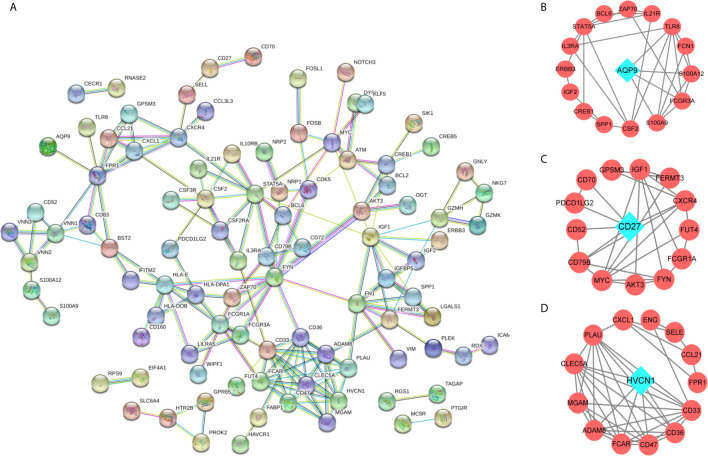
The protein-protein interaction (PPI) network for the DEGs. **(A)** PPI network for 166 immune-related DEGs, each round node represent a gene; **(B–D)** Sub-clusters of PPI network, the green diamond shape of node indicated the key gene of sub-cluster.

To identify key immune-related genes in CD, we screened DEGs between CD tissues and non-inflammatory tissues using the GSE95095 dataset. DEGs were defined as p-values less than 0.01 and 946 DEGs were identified. After overlapping DEGs and the immune-related genes, 117 immune-related DEGs were identified (**Supplementary Table S1**). By analyzing the GO functions of these immune-related DEGs, we observed that they were mainly involved in the cell activation and adhesion process, and the KEGG analysis revealed that cytokine receptor interaction pathways were the most enriched ones (**Supplementary Figure S2**). Using the PPI network, we constructed a network for the 117 immune-related DEGs, and three significant sub-clusters were identified, with each sub-cluster scoring 6.462, 4.154, and 4.133, respectively. *AQP9*, *CD27*, and *HVCN1* were the key genes of the three sub-clusters, respectively (**Figure 2**).

### Correlation of *AQP9*, *CD27*, and *HVCN1* With Immune Cells in CD

We noted that *AQP9*, *CD27*, and *HVCN1* were the cell markers of gamma delta T cells, activated B cells, and immature B cells, respectively, and the numbers of these immune cells were all increased in CD tissues compared with those in non-inflammatory tissues (**Figure S1**). Therefore, we examined the correlation of *AQP9*, *CD27*, and *HVCN1* with 28 immune cells in CD using the GSE95095 dataset. As shown in **Table 2**, *AQP9* was significantly correlated with Activated CD4 T cell, Activated CD8 T cell, Activated dendritic cell, Macrophage, Regulatory T cell, Type 17 T helper cell, Type 2 T helper cell (p < 0.05); *CD27* was remarkably correlated with Activated B cell, Central memory CD8 T cell, Effector memory CD4 T cell, Effector memory CD8 T cell, Natural killer cell, T follicular helper cell (p < 0.05); *HVCN1* was greatly correlated with Activated B cell, Effector memory CD8 T cell, Immature B cell, Natural killer cell, Neutrophil, T follicular helper cell, Type 1 T helper cell (p < 0.05).

**Table 2:** Correlation of AQP9, CD27, HVCN1 with immune cells in CD.

Immune cells types	CD27		AQP9		HVCN1	
	r	P	r	P	r	P
Activated B cell	0.376	0.008	-0.026	0.858	0.426	0.003
Activated CD4 T cell	-0.029	0.843	0.369	0.010	0.075	0.611
Activated CD8 T cell	0.244	0.095	-0.296	0.041	0.358	0.013
Activated dendritic cell	-0.123	0.404	0.475	0.001	-0.147	0.319
CD56bright natural killer cell	0.226	0.122	0.106	0.472	-0.178	0.227
CD56dim natural killer cell	-0.235	0.108	-0.056	0.706	-0.018	0.903
Central memory CD4 T cell	0.162	0.272	-0.056	0.704	0.103	0.488
Central memory CD8 T cell	0.331	0.021	0.107	0.469	0.230	0.115
Effector memory CD4 T cell	0.294	0.043	0.142	0.334	0.201	0.171
Effector memory CD8 T cell	0.457	0.001	-0.173	0.240	0.303	0.036
Eosinophil	0.047	0.753	-0.269	0.064	0.028	0.851
Gamma delta T cell	0.238	0.103	0.144	0.329	0.055	0.711
Immature B cell	0.125	0.397	-0.043	0.774	0.375	0.009
Immature dendritic cell	0.161	0.273	0.016	0.913	-0.243	0.096
Macrophage	-0.061	0.682	0.603	0.001	0.041	0.782
Mast cell	0.031	0.835	0.027	0.855	0.045	0.763
MDSC	0.403	0.005	0.440	0.002	0.262	0.072
Memory B cell	-0.073	0.622	0.033	0.826	0.205	0.161
Monocyte	-0.074	0.617	-0.093	0.528	0.085	0.568
Natural killer cell	0.367	0.010	0.210	0.152	0.287	0.048
Natural killer T cell	-0.074	0.618	0.222	0.130	-0.233	0.111
Neutrophil	-0.045	0.763	0.515	0.001	-0.287	0.048
Plasmacytoid dendritic cell	0.232	0.113	0.113	0.443	-0.029	0.846
Regulatory T cell	0.254	0.082	0.414	0.003	0.167	0.257
T follicular helper cell	0.386	0.007	-0.116	0.431	0.322	0.026
Type 1 T helper cell	0.204	0.164	0.176	0.231	0.349	0.015
Type 17 T helper cell	-0.099	0.505	0.317	0.028	-0.104	0.482
Type 2 T helper cell	-0.098	0.509	0.293	0.043	-0.022	0.884

### Diagnostic Values of *AQP9*, *CD27*, and *HVCN1* in CD

**Figure 3:**
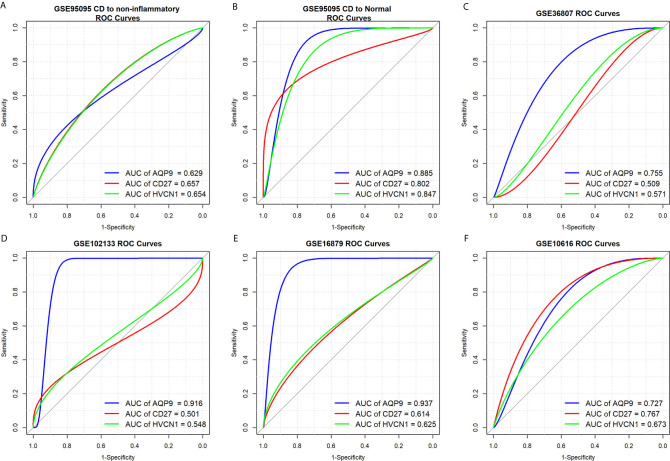
Diagnostic values of *AQP9*, *CD27* and *HVCN1* in CD. **(A)** Diagnostic value of *AQP9*, *CD27*, *HVCN1* in CD using non-inflammatory tissues as controls (GSE95095 dataset); **(B)** Diagnostic value of *AQP9*, *CD27*, *HVCN1* in CD using normal intestinal tissues as controls (GSE95095 dataset); **(C)** Diagnostic value of *AQP9*, *CD27*, *HVCN1* in CD (GSE36807 dataset); **(D)** Diagnostic value of *AQP9*, *CD27*, *HVCN1* in CD (GSE102133 dataset); **(E)** Diagnostic value of *AQP9*, *CD27*, *HVCN1* in CD (GSE16879 dataset); **(F)** Diagnostic value of *AQP9*, *CD27*, *HVCN1* in CD (GSE10616 dataset).

To determine the diagnostic value of *AQP9*, *CD27*, and *HVCN1* in CD, the GSE95095 dataset was first used as the training dataset. The results showed that when non-inflammatory tissues were used as controls, the AUC values of *AQP9*, *CD27*, and *HVCN1* in CD were 0629, 0.657, and 0.654, respectively. When normal intestinal tissues were used as controls, the AUC values of the above genes were 0.885, 0.802, and 0.847, respectively, indicating that these key genes from the PPI sub-clusters had a good performance in the diagnosis of CD, especially when using normal intestinal tissues as controls (**Figures 3A, B**).

Next, four datasets (GSE36807, GSE12366, GSE10616, and GSE102133) were employed to verify the results. The diagnostic value of the three genes was similar to the results from the GSE95095 dataset, and the diagnostic value for *AQP9* was better than that of *CD27*, and *HVCN1*; with the AUC value raised to 0.916 in the GSE1112366 dataset, and to 0.937 in the GSE16879 dataset (**Figures 3C–F**). These results indicated that AQP9 had a better diagnostic performance than *CD27* and *HVCN1* in CD.

### Predictive Values of *AQP9*, *CD27*, and *HVCN1* in the Treatment of CD

**Figure 4:**
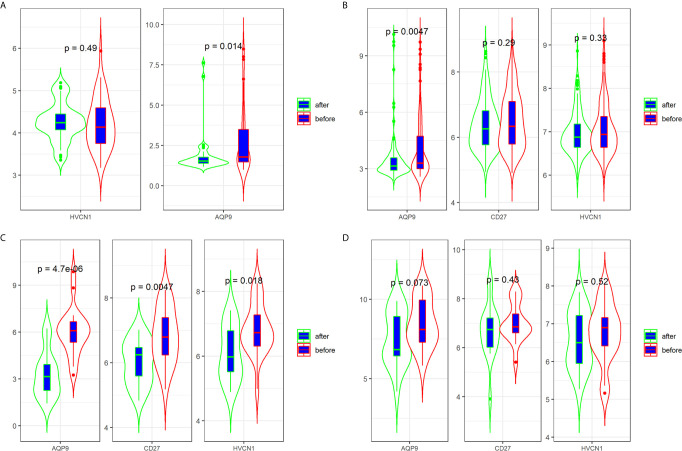
Expression of *AQP9*, *CD27* and *HVCN1* in CD tissues that underwent anti-TNFα therapy **(A)** Expression of *AQP9*, *CD27* and HVCN1 in GSE98820 (this dataset do not has *CD27* gene); **(B)** Expression of *AQP9*, CD27 and HVCN1 in GSE112366; **(C)** Expression of *AQP9*, *CD27* and *HVCN1* in GSE16879 (only selected responders); **(D)** Expression of *AQP9*, *CD27* and *HVCN1* in GSE16879 (only selected non-responders).

Three datasets (GSE16879, GSE98820, and GSE112366) provided the data for patients with CD who had undergone anti-TNFα therapy. Specifically, the GSE16879 dataset provided the data before and after the first infliximab treatment, and the response or no response data to the treatment; GSE98820 dataset provided the data for adalimumab treatment; GSE112366 dataset provided the data for ustekinumab treatment. As shown in GSE98820 and GSE112366 datasets, all patients had responded to anti-TNFα therapy. The results showed that only the expression of AQP9 was decreased after treatment with CD in the three datasets (**Figures 4A–C**). In particular, the expression of AQP9 did not significantly change in non-responders (p = 0.072; **Figure 4D**).

**Figure 5:**
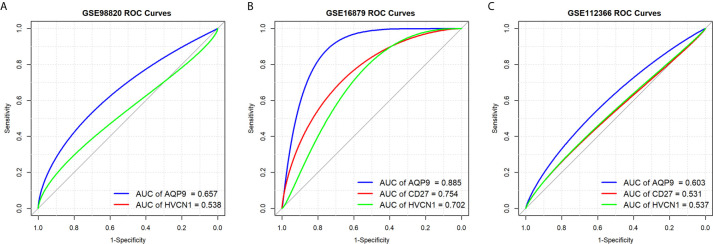
Predictive values of *AQP9*, *CD27* and *HVCN1* in the treatment of CD. **(A)** Predictive value of *AQP9* and *HVCN1* in the treatment of CD (GSE98820); **(B)** Predictive value of *AQP9*, *CD27* and *HVCN1* in the treatment of CD (GSE16879); **(C)** Predictive value of *AQP9*, *CD27* and *HVCN1* in the treatment of CD (GSE112366).

Next, we determined the predictive value of *AQP9*, *CD27*, and *HVCN1* in the treatment of CD, as shown in **Figure 5**; *AQP9* had a moderate predictive value in patients who had undergone treatment (AUC of *AQP9*: 0.603–0.885), while the predictive value of *CD27* and *HVCN1* was mild (AUC of *CD27*: 0.531–0.754; AUC of *HVCN1*: 0.537–0.702). Taken together, these results indicated that *AQP9* was a promising marker for monitoring and predicting the treatment effect in CD.

### Expression of AQP9, CD27, and HVCN1 in CD Tissues

**Figure 6:**
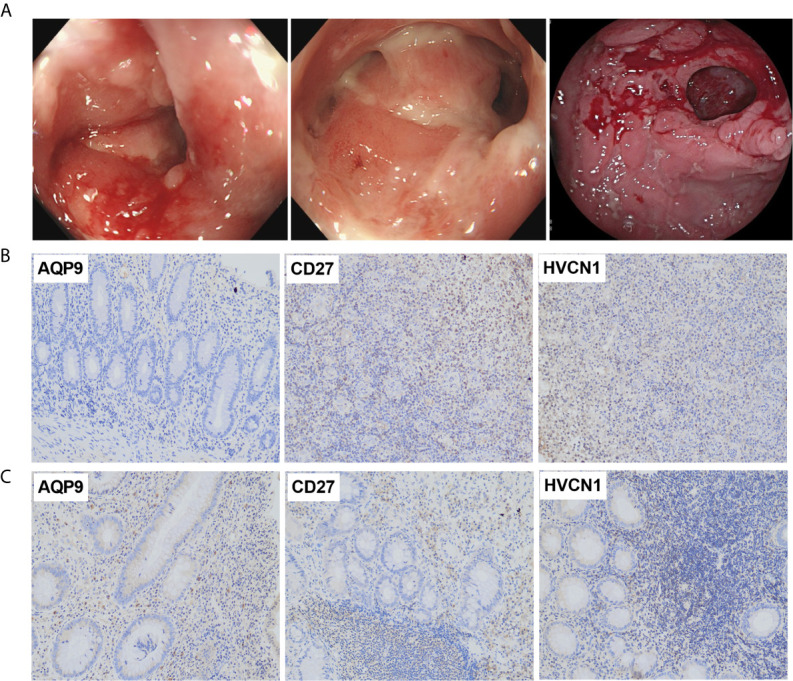
**(A)** Typical endoscopic images of CD: left, ileum in remission; middle, ileocecal valve; right, colon with ulcer; **(B)** Expression of *AQP9*, *CD27* and *HVCN1* in adjacent normal intestinal tissues, fewer immunostaining can be seen in the mucosa (Magnification ×200); **(C)** Expression of *AQP9*, *CD27* and *HVCN1* in CD tissues, more immunostaining can be seen in the mucosa (Magnification ×200).

**Figure 7:**
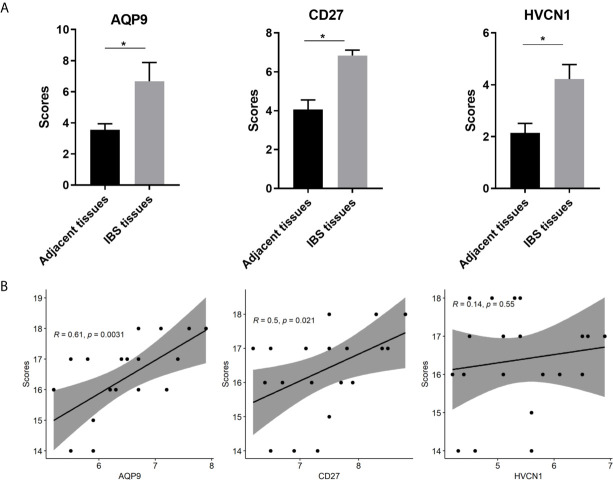
**(A)** Comparison of IHC intensity score between adjacent normal intestinal tissues and CD tissues regarding the *AQP9*, *CD27* and *HVCN1*; **(B)** Correlation analysis between the CDAI scores and the expression of *AQP9*, *CD27* and *HVCN1* in CD tissues. IHC, Immunohistochemical; CDAI, Crohn’s Disease Activity Index, *indicated p-value < 0.05.

The demographic and endoscopic data was listed in the **Table 3** and **Figure 6A**, which the CD was at the activated stage, with the Crohn’s Disease Activity Index (CDAI) ranged from 9 to 16 scores. Using CD tissues from our hospital, we detected the expression of AQP9, CD27, and HVCN1 *via* IHC (**Figures 6B, C**). The results revealed that the expression of AQP9, CD27, and HVCN1 was considerably increased in CD tissues compared with the corresponding normal intestinal tissues (p < 0.05), which was consistent with the results of the GSE95095 dataset. We also assessed the association of these genes with the clinical features of patients with CD. However, none of the genes was associated with the patient’s age, gender, and smoking status (p > 0.05; **Figure 7A**). The correlation analysis revealed that there were significant correlations between the CDAI with *AQP9* and *CD27*, respectively, but not with *HVCN1*, suggesting *AQP9* and *CD27* were also associated with the activity of CD (**Figure 7B**). Taken together, our results suggested that *AQP9*, *CD27*, and *HVCN1*, were involved in the pathogenesis of CD.

**Table 3:** Demographic data of CD patients.

Variables	Data
Age (years)	33.5 ± 10.8
Gender (n)	
Male	12
Female	8
Smoking (n)	3
Location	
Ileocecal	6
Ileum	8
Ascending colon	3
Descending colon	3
CDAI (score)	16.7 ± 2.4

CDAI, Crohn’s Disease Activity Index.

## Discussion

Evidences have confirmed that CD is a chronic inflammatory intestinal disease, which is caused by an unrestrainable immune response against luminal bacterial antigens. The role of inflammatory cells infiltrating in maintaining an active stage is well established and most of the therapeutic strategies are aiming to block the cascade of inflammatory reaction and release of pro-inflammatory cytokines (14, 15). In the present study, by analyzing the data for CD, non-inflammatory tissues, and normal intestinal tissues using ssGSEA, we described the landscape of immune infiltration in CD and found that the fraction of 16 immune cells was significantly different among these three kinds of tissues. In addition, we found more immune cells that were significantly different between CD tissues and non-inflammatory tissues, and most of the immune cells showed higher infiltration in CD tissues than in non-inflammatory tissues. These results were consistent with previous studies (9), which further confirmed the important role of abnormal immune cells in the pathogenesis and progression of CD.

The abnormal immune system is a critical contributor to the pathogenesis and progression of CD (16). During the last decades, many studies have confirmed that CD tissues often present high immune infiltration, and immune cells and immune-related genes are attractive targets for modulating CD progression (17). Importantly, innate and adaptive immune cell infiltration is closely associated with patient response to treatments and clinical outcomes. Therefore, this evidence indicates that the application of immune-based features in CD is a reasonable approach to estimate the pathogenesis and clinical outcome (18). By analyzing the transcriptome data of CD using ssGSEA (19), xCell (20) or CIBERSORTx (9), researchers could provide insights into the regulatory network between CD and immune cells, which could deepen our knowledge on the association of CD with its immune environment.

Generally, immune cells perform their role by secreting immune-related cytokines. We screened the potential immune-related genes by overlapping the immune cell markers and DEGs and using a PPI network to identify the key immune-related genes. The PPI network and the sub-cluster analysis method have been widely applied to identify key genes in the pathogenesis of diseases (21, 22). Using the same method, we firstly established a PPI for the immune-related genes in CD, and then screened the key genes from the sub-cluster of PPI. The results showed that *AQP9, CD27*, and *HVCN1* ranked the highest score in each sub-cluster, and the correlation analysis demonstrated that these three genes were significantly correlated with several immune cells. It is noteworthy that *AQP9, CD27*, and *HVCN1* are associated with CD, as shown in previous studies. For example, *AQP9* in the peripheral blood could discriminate patients with CD from patients with chronic inflammation and healthy controls (23). In rat myocardial infarction model, the mRNA and protein expression levels of *AQP9* was significantly increased, and silencing *AQP9* gene can inhibit the activation of ERK1/2 signaling pathway, attenuate the inflammatory response in rats with myocardial infarction, inhibit apoptosis of myocardial cells, and improve cardiac function (24). In addition, *AQP9* was showed to play a role in regulating tissue-specific physiological properties in tight junctions in ulcerative colitis (25). Regarding the CD27 in CD, reduced CD27^-^ IgD^-^ B cells in the blood and raised CD27^-^ IgD^-^ B cells was found in the gut-associated lymphoid tissue in IBD (26), study also observed that patients with CD were characterized by a reduced proportion of B cells of the memory CD27+ phenotype compared to the non-IBD controls (27). In term of *HVCN1*, expression of *HVCN1* was found to significantly positively correlate with the clinical activity of CD (28). Moreover, in cystic fibrosis patients who underwent ivacaftor therapy, the *HVCN1* mRNA expression was significantly higher than baseline at 1-3 months and decreased after 6 months of treatment (29). The similar results were found in cystic fibrosis patients who underwent antibiotic therapy (30). All the above evidences demonstrated that the three genes play critical roles in the pathogenesis of inflammatory diseases or CD. Therefore, we select these three genes in the following analysis.

Since *AQP9, CD27*, and *HVCN1* are immune-related genes overexpressed in CD tissues, we assessed the diagnostic value of these three genes. As expected, *AQP9, CD27*, and *HVCN1* had a mild to moderate diagnostic value in CD, especially when using normal intestinal tissues as controls, and the diagnostic value of *AQP9* was much better than that of *CD27* and *HVCN1*. Although the diagnostic value of *CD27* has been reported in multiple myeloma (31), its diagnostic value in CD has not been described previously. It should be noted that Chen et al. (9) showed that *CXCL8*, encoding IL-1B, together with immune cells has a good diagnostic value in CD. Therefore, our results provided other candidate diagnostic indicators for diagnosis.

Regarding the treatment of CD, infliximab, adalimumab, and ustekinumab are the crucial agents of anti-TNFα therapy, which have shown high efficacy in many patients with CD (32, 33). However, early identification of non-responders to anti-TNFα therapy remains a challenge in the clinical setting. Currently, some indicators or methods have been reported to predict the response of patients with CD who underwent anti-TNFα therapy, such as serum oncostatin M (34), small bowel ultrasound (35), and SMAD7 (36). However, more indicators still need to be explored. In this study, we examined the predictive value of *AQP9, CD27*, and *HVCN1* in patients who underwent anti-TNFα therapy. Our results indicated that only *AQP9* had a good predictive value in those responders. More importantly, the expression of AQP9 did not significantly decrease in non-responders, suggesting a higher specificity of AQP9. To date, although some inflammatory and immune biomarkers are used to diagnose the CD, considering the difficulty of diagnosis CD in the clinical setting, this study provided other alternatives to the diagnosis of CD, and these biomarkers also help to assess the anti-TNFα therapy on CD patients. In addition, our results imply that the expression of *AQP9* and *CD27*, was significantly correlated to the activity of CD, and *AQP9* could be served as a valuable indicator to monitor the effect of anti-TNFα treatment.

Although the present study employed many GEO datasets with a larger number of tissues to explore the immune cells and immune-related genes in CD and identified three genes, with promising diagnostic and predictive values in patients with CD, some limitations remain to be addressed. First, the fraction of immune cells in CD tissues was analyzed using ssGSEA; therefore, the results need to be verified using tissue-based flow cytometry. Second, we applied IHC to validate the expression of AQP9, CD27, and HVCN1 in CD tissues; however, due to lack of samples after anti-TNFα therapy, the predictive ability of these genes still needs to be validated in future studies. Third, due to the lack of data, including disease phenotype and disease activity status, the associations between immune cells and related genes, and disease severity, need to be further estimated.

## Conclusions

The present study provided insight into the landscape of immune cells underlying CD and identified *AQP9, CD27*, and *HVCN1* as auxiliary diagnostic indicators for CD. We also identified *AQP9* as a promising predictive indicator in patients who underwent anti-TNF therapy. However, future studies with larger samples and clinical information using flow cytometry analyses are warranted to validate these findings.

## Data Availability Statement

The raw data supporting the conclusions of this article will be made available by the authors, without undue reservation.

## Ethics Statement

The studies involving human participants were reviewed and approved by People’s Hospital of Guangxi Zhuang Autonomous Region. The patients/participants provided their written informed consent to participate in this study.

## Author Contributions 

Study concept and design: BY, X-wG, and B-lH. Collection and assembly of data: Y-xY, Z-gP, and Y-pT. Data analysis and interpretation: BY, Y-xY, Z-gP, and Y-pT. Immunohistochemical assay: BY, K-lW, and X-wG. Manuscript revised: B-lH, K-ZL, and BY. Manuscript writing and review: All authors. All authors contributed to the article and approved the submitted version.

## Funding

This study was partially supported by research funding from Cultivation project of NSFC (no. GJPY2019005) and the National Natural Science Foundation of Guangxi (no. 2020GXNSFAA297116).

## Conflict of Interest

The authors declare that the research was conducted in the absence of any commercial or financial relationships that could be construed as a potential conflict of interest.
