# Supplementary material for: Diagnostic and Predictive Value of Immune-Related Genes in Crohn’s Disease
Source: Front Immunol. 2021 Apr 16;12:643036. doi: 10.3389/fimmu.2021.643036 (PMC8085323; doi:10.3389/fimmu.2021.643036)
Supplement: Supplementary file 1 [file DataSheet_1.pdf]

Supplemental figures

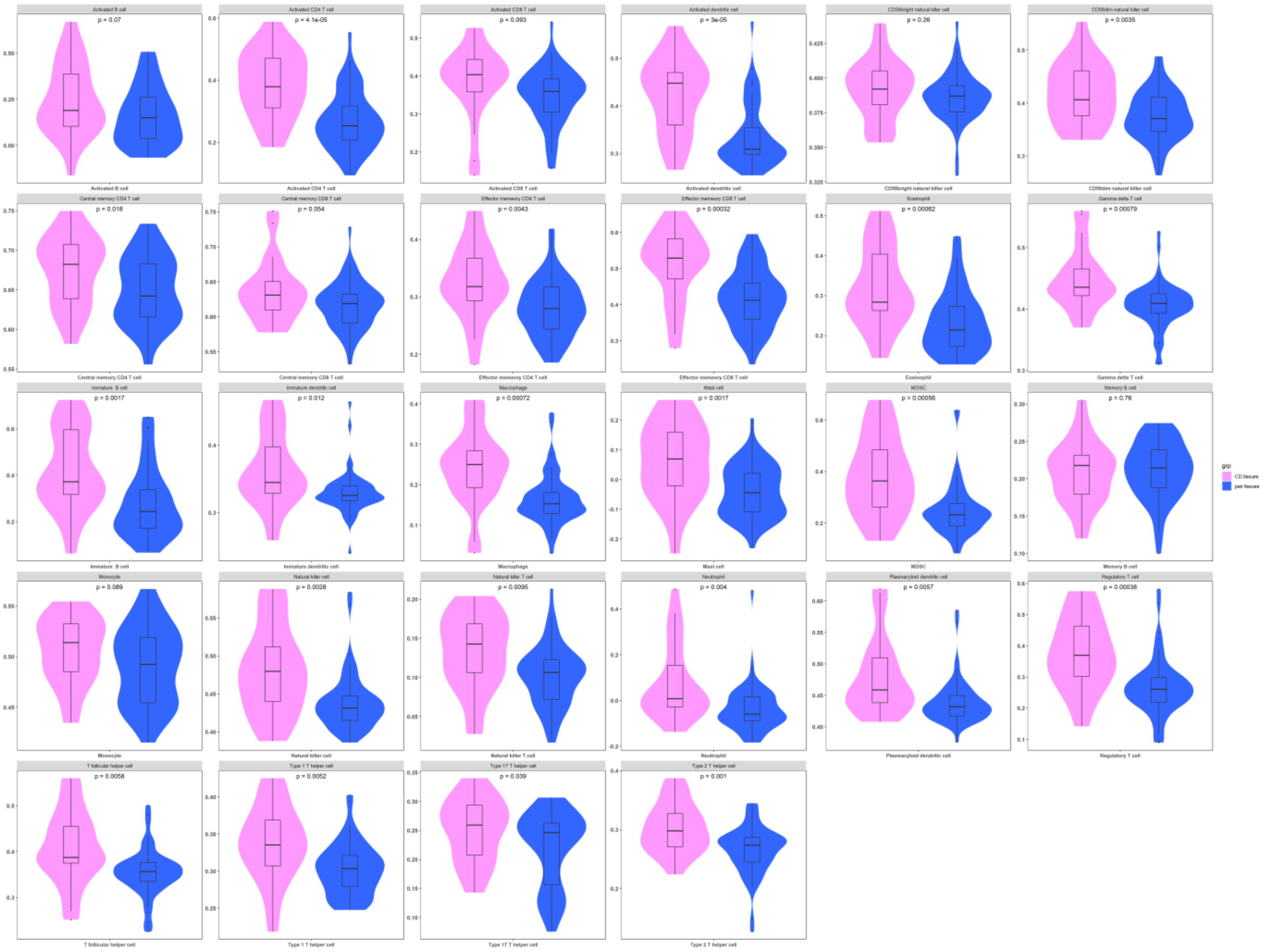

Figure S1 Comparison of 28 immune cells between CD tissues and non-inflammatory tissues

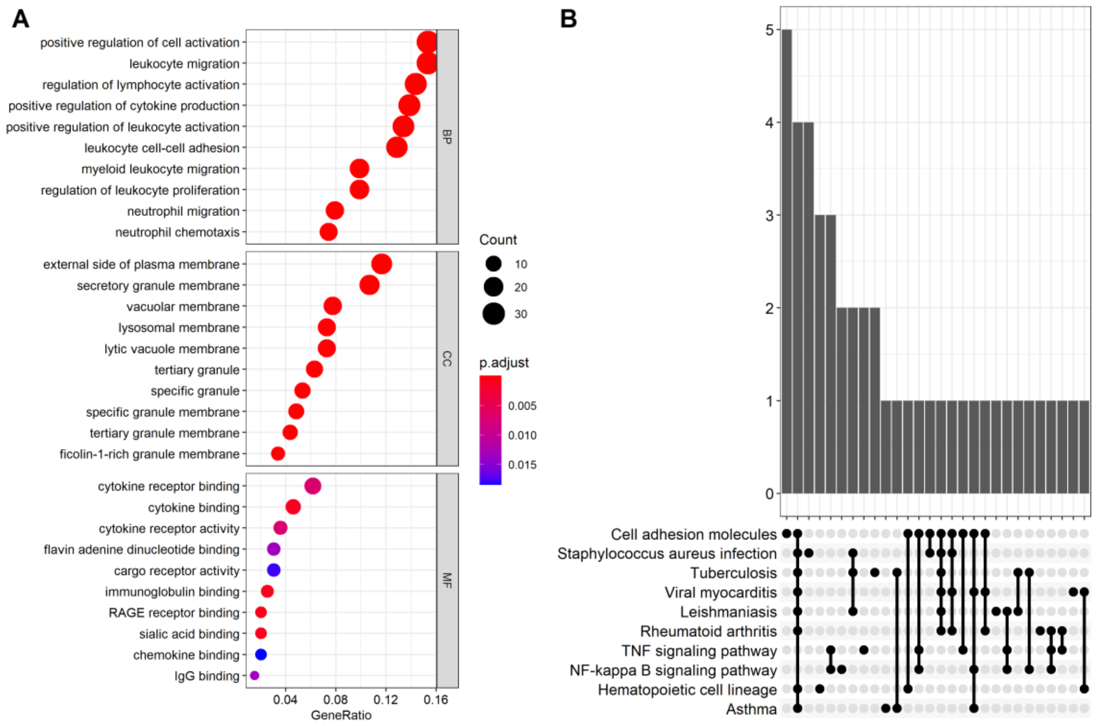

Figure S2 Function enrichment of immune-related different expression genes (DEGs). (A) GO function of immune-related DEGs, including Biological Process (BP), Molecular Function (MF), and Cellular Component (CC); (B) KEGG analysis of the immune-related DEGs.
